# Non-Contact Video-Based Assessment of the Respiratory Function Using a RGB-D Camera

**DOI:** 10.3390/s21165605

**Published:** 2021-08-20

**Authors:** Andrea Valenzuela, Nicolás Sibuet, Gemma Hornero, Oscar Casas

**Affiliations:** Instrumentation, Sensors and Interfaces Group, Universitat Politècnica de Catalunya, Carrer de Jordi Girona, 1, 3, 08034 Barcelona, Spain; aand.valenzuela@gmail.com (A.V.); nicolas.sr.22@gmail.com (N.S.); gemma.hornero@upc.edu (G.H.)

**Keywords:** breath rate, contactless assessment, healthcare monitoring, non-contact respiratory rate measurement

## Abstract

A fully automatic, non-contact method for the assessment of the respiratory function is proposed using an RGB-D camera-based technology. The proposed algorithm relies on the depth channel of the camera to estimate the movements of the body’s trunk during breathing. It solves in fixed-time complexity, O(1), as the acquisition relies on the mean depth value of the target regions only using the color channels to automatically locate them. This simplicity allows the extraction of real-time values of the respiration, as well as the synchronous assessment on multiple body parts. Two different experiments have been performed: a first one conducted on 10 users in a single region and with a fixed breathing frequency, and a second one conducted on 20 users considering a simultaneous acquisition in two regions. The breath rate has then been computed and compared with a reference measurement. The results show a non-statistically significant bias of 0.11 breaths/min and 96% limits of agreement of −2.21/2.34 breaths/min regarding the breath-by-breath assessment. The overall real-time assessment shows a RMSE of 0.21 breaths/min. We have shown that this method is suitable for applications where respiration needs to be monitored in non-ambulatory and static environments.

## 1. Introduction

Advances in healthcare are pointing towards the development of systems allowing the remote monitoring of the person in its everyday life [1,2]. Among the different physiological signals that can be ambulatory tracked, the respiration signal can provide an insight into a person’s general state of health [3,4]. In addition, its continuous supervision could be used as a method to detect future diseases due to the subtle changes in the respiratory function that common diseases present prior to the onset of clinical symptoms [5]. The breath rate (BR) has been found to be a more discriminating parameter between stable and unstable patients than the heart rate (HR) [6].

The monitoring of the respiratory signal could be used to detect and control respiratory diseases such as chronic obstructive pulmonary disease, which is considered one of the most common long-term conditions; hypoxemia or hypercarbia; sleeping disorders; or prediction of cardiac arrest [6]; among others. Even in the COVID-19 pandemic, the detection of changes in the BR are of critical interest in early infections due to the severe effects that the virus can have on the lungs. The virus causes a lower respiratory tract infection in most cases reducing the overall efficiency of the lungs, which results in an increase in the BR [7]. A significant increase is not as common as in other cases of viral illnesses such as influenza or common cold, among others, as these viruses typically affect the upper respiratory tract [8].

There are some respiratory disorders in which one needs to track the respiration signal from different parts of the body. The most common ones need the analysis of the phase relation between thoracic and abdominal respiration signals [9]. The detection of this phase is useful to identify and control, for example, sleep apnea syndrome (SAS), a very common sleep disorder [10]. An episode of apnea occurs if someone’s breathing ceases totally during sleep for more than 10 s [11]. Episodes of central apnea (CA) and obstructive apnea (OA) can be distinguished by analyzing the respiration movements jointly at both the thorax and abdomen. In the case of CA, the movements have very low amplitudes compared to normal breathing while, during an OA, the obstruction of the airways leads to an increase in the respiratory movements trying to overwhelm the obstruction [10]. In these cases, a multi-point assessment of both thoracic and abdominal regions needs to be performed.

There are several ways for measuring the respiration signal that, according to the authors of [12], could be classified as follows:

1. Methods to extract the respiratory signal from other physiological signals that are modulated by respiration: electrocardiography, photoplethysmography, and electrical impedance measurements have been used, among others [13,14,15].
2. Methods based on volume changes and body movements: during inhalation and exhalation, there are periodic volume changes in the thoracic and abdominal areas that are manifested by movements on the body surface. These changes could be detected by accelerometers [16], gyrometers [17], by measuring the force applied on a band by chest or abdominal expansion [18,19], by using acceleration and force sensing modules, or even highly sensitive fiber optic attached to a mattress [20]. Other sensors based on electromagnetic, piezoresistive, and piezoelectric mechanisms have been also used [21].
3. Methods based on air flow changes, as the respiratory airflow causes various effects around the nasal area the periodic fluctuations in temperature, humidity, the density of carbon dioxide, and even the respiratory sounds have been used [22].

Regarding the measurements based on volume changes and body movements, all of the aforementioned examples require direct contact with the user and additional pose requirements, making them uncomfortable and not accessible to everyone [23]. A novel approach to the respiratory function assessment is the non-contact measurements of the respiratory motion. Non-contact measurements are more comfortable for the patient, but especially in the cases where contact measurements are especially difficult to perform: contactless monitoring of neonates is highly desired due to their fragile skin or in people who have suffered body burns [24,25]. These new non-contact assessments are being applied also in non-ambulatory environments. For example, the automobile industry is calling for the improvement of road security detecting drowsiness in drivers [21], general sleep monitoring systems [26], or even in long-term condition patients, bringing healthcare tracking at home [27]. Rising living standards are causing people to live longer, but sometimes with more than one long-term medical condition that must be under continuous supervision [28].

Lately, the new contactless methods for the assessment of the respiratory motion are mainly based on video analysis with traditional color video cameras (RGB) [3], depth cameras (RGB-D) [29], infrared (IR) cameras [30], lidars and radars [24,31,32], or even WiFi devices [33]. Regarding the algorithms used for the recovery of the respiratory signal, the vast majority are based on variations of the intensity of the acquired signal [34], computations of the Power Spectral Density (PSD) [35], Principal Component Analysis (PCA) on the RGB channels of the cameras [36,37] or by using using optical flow and the natural pattern of the flow vectors that results in their convergence and divergence during breathing [24,38]. However, those processing algorithms are so complex that almost none of them can rely either on breath-by-breath monitoring in the real-time domain or in the synchronous measurement in different parts of the body.

This article revolves around the development of an acquisition and processing algorithm capable of retrieving the real-time assessment of the respiration signal from multiple parts of the body, synchronously and without physical contact by using the depth channel of a low-cost RGB-D camera.

Concretely, the device used for the acquisition of the signal is the Intel RealSense Depth Camera D435 [39] that, besides the traditional color image video, outputs an additional video signal where each pixel value represents its depth in the real world. This type of Intel camera has a depth accuracy below 1% of the distance to the object. Therefore, if the camera is 1 m away from the object, the expected sensitivity for measuring changes is between 2.5 mm and 5 mm, which gives to the user a pretty good estimation of the real-world distances. In general, 3D cameras provide many advantages over 2D cameras—RGB channels only—as depth information can be used for more accurate detection of the regions of interest (ROIs) and robust motion measurements [40].

Both the real-time and multi-point assessment are achieved thanks to the simplicity of the proposed algorithm that only relies on the mean value of the depth signal to estimate the movement of the ROIs with respect to the camera, achieving fixed-time complexity, O(1), and getting rid of time-consuming processing of the traditional RGB channels. Instead, the color channels are used to automatically locate the ROIs in which the depth analysis is lately performed. Therefore, the main contributions of this work are as follows:

- Automatic location of the body parts in which the measure is going to be performed.
- Fixed time-complexity algorithm that solves in real time providing reliable breath-by-breath information.
- Synchronous multi-point assessment of the respiratory signal.

Although the non-contact breathing monitoring by means of the depth information has been already proposed in other studies, the proposed method overcomes the state-of-the-art error rates in multiple body regions that are automatically located, it targets the acquisition of the complete respiratory function, it provides real-time assessment thanks to the simplicity of the processing algorithm, and it has been widely validated considering users with a wide age range and different bodies and health conditions.

## 2. Materials and Methods

### 2.1. Face Detection and ROI

As already mentioned, the proposed method relies on the RGB channels of the camera to locate the user’s face and automatically compute the ROIs according to the physiology of the user. This step makes the non-contact assessment even more flexible and comfortable as the algorithm is able to locate the ROIs independently of the position of the user and distance to the camera, within a certain range. To do so, the initial position of the user’s face in the frames captured by the camera should be identified, without having any past position reference. There are several techniques available to identify and segment objects in images. In particular, for the detection of faces, the most popular ones are the Viola–Jones algorithm [41], methods based on Histogram of Oriented Gradients (HOG) [42], and deep learning methods mainly based on Convolutional Neural Networks (CNN) [43,44,45].

From these three, CNN-based methods are considered the state-of-the-art as they are able to recognize faces in almost any condition of pose, rotation, size, and illumination. However, they are computationally expensive unless using specialized hardware. The other two methods are faster and relatively lightweight and they could be easily used in real-time. Among these two, the Viola–Jones method has a higher success rate than HOG, meaning that it is capable of correctly detecting faces more frequently than HOG [46]. However, it is also more prone to obtain false positives [47]. In this case, the implementation of the Viola–Jones algorithm from the OpenCV library [48] is used along with a preprocessing routine to reduce this probability of false detection.

To reduce these probabilities of false detection from the Viola–Jones algorithm, the regions of the color image with depth values over 1.5 m are masked. Those with null depth value are also masked as they are noisy components. After that, a closing morphological operation [49] is also applied on the mask to get rid of the non-masked holes that are sometimes left in the image. This operation consists on dilating the unmasked zones closing any interior hole and then eroding to restore the initial contour of the unmasked objects. As a result, all the background of the image is masked, letting the person alone in the scene in which the face detection algorithm is applied.

**Figure 1:**
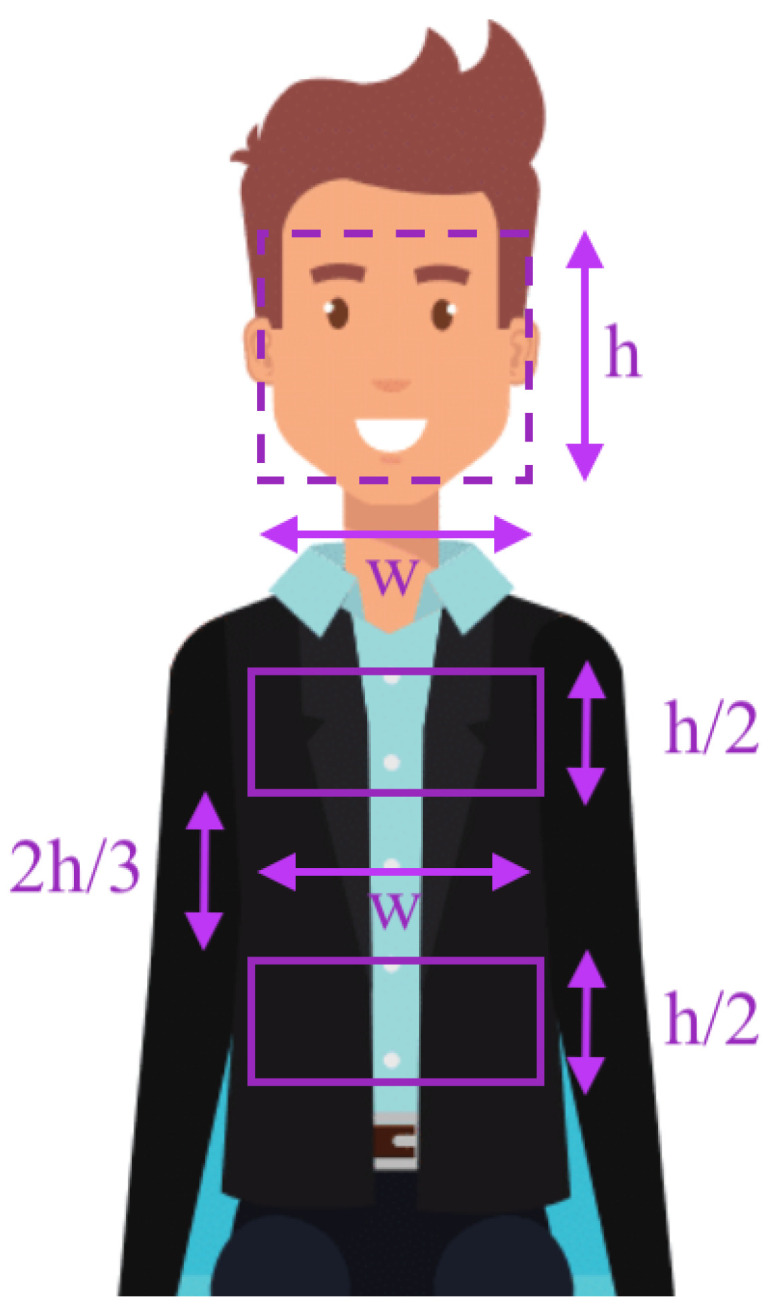
Automatic location of the thoracic and abdominal regions based on the position of the face. The face of the user is detected through the Viola-Jones algorithm.

The face detection is then performed using the Viola–Jones algorithm on the color stream. If the algorithm fails to detect the face in the first frame, it searches for a face in the following frames until the face region is detected. At this point, it is interesting to note that the algorithm searches for the user’s face in the whole image, so there is no strict condition on the location of the user in the image, as long as the face and the ROIs are visible. Once the face is successfully detected, a boundary box around is obtained. The dimensions and position of this bounding box are then used to compute the ROIs in which the respiratory signal is going to be acquired. Therefore, the adaptation of the detection algorithm to the physiology of the user is ensured. In order to define the ROIs, it is important to consider the resolution of the output image. In our case, the resolution of the frames is 640×480, meaning that each frame could be seen as a 640×480 matrix whose upper-left vertex corresponds to the point (0,0) while its lower-right vertex corresponds to the point (640,480). The detection algorithm returns the position of the face in the form of the $\left(x_{face},y_{face}\right)$ coordinates that correspond to the upper-left vertex of the bounding box and both the height, $h_{face}$, and the width, $w_{face}$, of the face. From those values, the ROIs have been mathematically defined in coordinates as

(1) $$\left(x_{thorax},y_{thorax}\right)=\left(x_{face},\;480-2h_{face}\right)$$

for the upper-left vertex of the thorax, and

(2) $$\left(x_{thorax},y_{thorax}\right)=\left(x_{face}+w_{face},\;480-\frac{3h_{face}}{2}\right)$$

for its lower-right vertex. Analogously, the ROI of the abdomen has been defined as:

(3) $$\left(x_{abdomen},y_{abdomen}\right)=\left(x_{face},\;480-\frac{5h_{face}}{6}\right)$$

for its the upper-left vertex, and

(4) $$\left(x_{abdomen},y_{abdomen}\right)=\left(x_{face}+w_{face},\;480-\frac{h_{face}}{3}\right)$$

for its lower-right vertex. Therefore, the ROIs are defined as a rectangle of area $w\frac{h}{2}$. Figure 1 depicts a diagram of the automatic location of those regions based on the detected position of the face. In general, we have avoided the usage of ROIs of fixed area, and independent of the body shape, in order to make the algorithm suitable for any participant at any distance to the camera. Once the ROIs are defined, they do not change in an entire cycle. Finally, location changes of the users during the measurements, e.g., walking users, are out of the scope of the present work as no face or ROI tracking is continuously performed on the RGB channel.

The ROIs equations have been defined by taking different video samples recorded with the same acquisition device and by checking the location of the thoracic and the abdominal regions with respect of the position of the face. Those videos included participants with different genders, body shapes and an age range between 23 and 52 years old. Lately, the validation of the proposed equations was performed with the video samples of the experiments. Note that the mean depth value of the pixels encapsulated within the ROI is then used for the assessment of the signal and that the whole body’s trunk experiences respiration movements. Therefore, although these equations have been proved to provide the smallest error in the experiments, the effect of slightly modifying the ROI in size or in position—because of both face size and body shape—has not been shown to have a direct impact on the acquired signal. In addition, these ROIs have been shown to be adaptable in a range distance up to 2 m with respect to the camera. Nevertheless, for the normal operational mode of the acquisition system, the background mask is applied at a distance of 1.5 m with respect to the camera.

### 2.2. Signal Acquisition and Processing

From this point onward, once the ROIs have been selected, the mean of the depth value of all the pixels inside each ROI is computed and saved at each frame along with the timestamp for the current frame. This raw signal, although noisy, already reflects the intake and outtake of air when breathing. The signal is first normalized, and its mean is set to zero to reduce transitory states when filtering later. The filter itself is a moving-average filter commonly used for smoothing noisy data. Equation (5) defines the moving-average filter on our raw signal *x* after the normalization:

(5) $$y\left(n\right)=\frac{1}{ws}\cdot \left(x\left(n\right)+x\left(n-1\right)+...+x\left(n-\left(ws-1\right)\right)\right)$$

where *ws* is the window size, *x* is the input raw data, and *y* is the smooth output signal.

Concretely, the averages have been computed along the data vector every 8 samples. The length of the window applied has been adjusted considering a good trade-off between a smooth output signal and not displacing the maximum and minimum values of the signal along the timestamp. Finally, the slightly averaged signal is passed through a bandpass Butterworth filter with a high cut-off frequency of 0.5 Hz and a low cut-off frequency of 0.08 Hz. The cut-off frequencies correspond to 30 bpm and 4.8 bpm, respectively. As a reference, the normal BR for people over 7 years old ranges from 12 to 20 bpm [30]. Again, the resulting signal is a smoother one that maintains the time location of the breathing peaks and troughs of the original signal. At this point, the parameters of interest that are commonly used to track the respiration signal could be computed. For example, the BR can be computed from the acquired signal from either the time between consecutive peaks or the time between consecutive troughs. The frame rate of the camera has been set to 15 fps as a good trade-off between a stable frame rate and a sufficient number of samples to reproduce the signal. With the selected frame rate, the depth module of the camera resolves one sample every 0.0667 s, on average. Note that the most time-consuming routines of user’s face location and computation of the ROIs are performed with the RGB channels aside from the main execution of the depth value acquisition. Therefore, the actual time of computing the mean of the ROI and filtering (O(1)) is negligible with respect to the intrinsic delay of 0.0667 s introduced by the frame rate.

**Figure 2:**
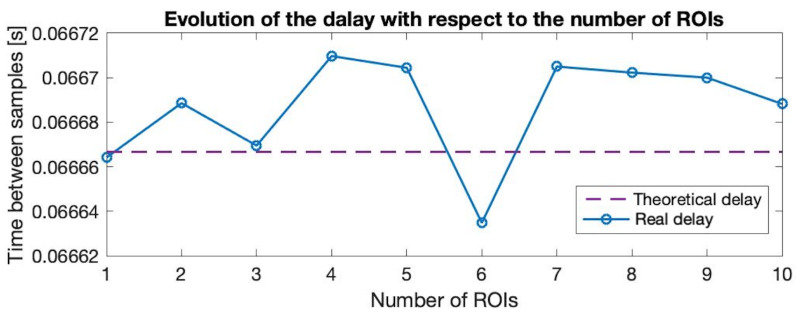
Evolution of the time between consecutive samples when increasing the number of ROIs from 1 up to 10. The results show that the computation of the mean depth value in the different regions of the image do not introduce a significant delay in the normal time between samples. The difference between the theoretical time and the real-time between consecutive samples is not different from the intrinsic error of the camera.

Figure 2 displays the mean time between samples averaged in three different runs considering an increasing number of ROIs. It shows that the addition of more mean depth value computations in other regions of the image does not impact the time interval between one sample and its consecutive. We have proved that increasing the number of ROIs up to 10 regions does not impact neither in the time interval between samples nor in the time complexity of the algorithm, but allows a synchronous multi-point assessment. In addition, variations on the size of the ROIs do not introduce a significant delay in the acquisition of samples.

**Figure 3:**
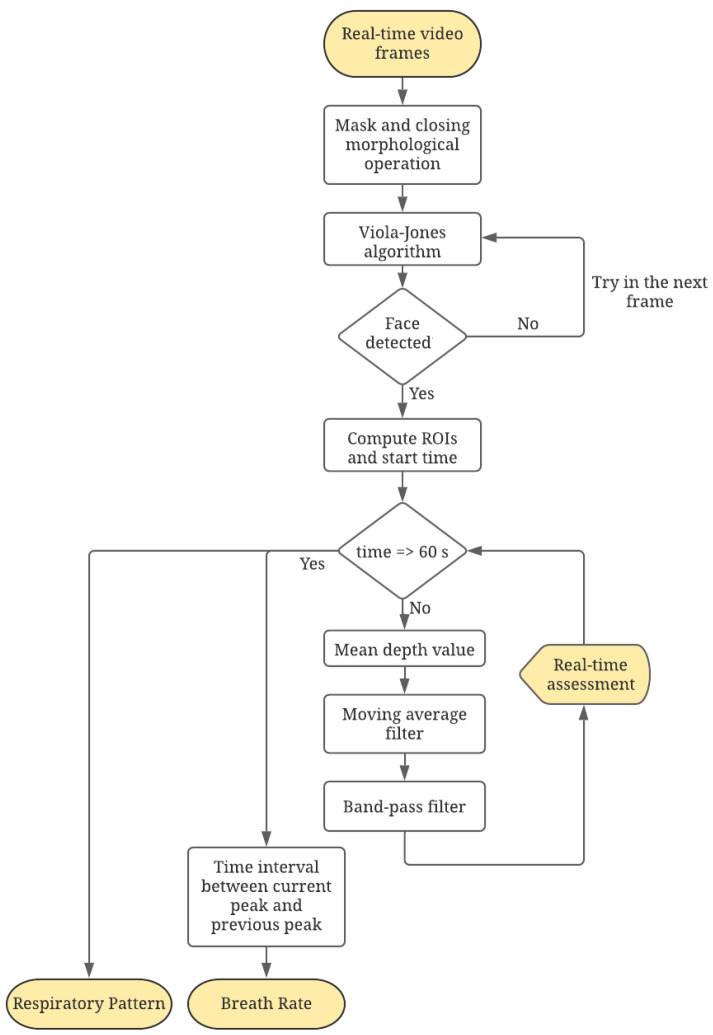
Flow chart of the proposed algorithm.

Figure 3 shows the architecture of the proposed algorithm for the acquisition and processing of the respiratory signal. The implementation of both the acquisition and the processing routines have been implemented in Python 3.6 while the statistical analysis of the results has been implemented in MATLABR_2020a.

## 3. Results

To validate the developed software, two different experiments have been performed on 10 and 20 different voluntary users, respectively. The first one aims to show the performance and validity of the proposed algorithm in different scenarios, body poses, body types (variable sizes and skin tones), physical conditions, and clothing, but always with the user in a non-moving position. In order to maximize its potential use, users with cerebral palsy who have uncontrolled movements have been included. The second one aims to closely compare the obtained signal with a reference one to accurately evaluate the performance of the algorithm. When in any of the two experiments, due to incorrect positioning of the reference sensor on the abdomen or motion artifacts affecting the camera, it was not possible to obtain the respiratory rate in a comparative manner, the results were reflected with a dash. In this way, the percentage of correct functioning of the system is also presented.

### 3.1. First Experiment: Single ROI

A first set of 10 users, namely, U1-10, were selected to validate the proposed algorithm by acquiring the respiration signal in one single thoracic region. The users were told to synchronize their breathing to a fixed—and known—frequency by using a simple metronome. The ages of the participants ranged from 21 to 66 years old with an average age of 34 years. All the participants provided their explicit consent. Each user was invited to stay at a mean distance about 1 m to the acquisition device. They were asked to face the camera—but not necessary looking directly towards it, remain still, and breathe according to the metronome during the measurements. The respiratory frequency tested was chosen randomly within the range of 12 to 25 bpm, making sure first that the selected rhythm was comfortable for each user. All the measurements were carried out indoors, but in different scenarios with a stable amount of light and during 60 s each. Nevertheless, as the acquisition itself relies on the depth module of the camera, no specific light conditions are required. Finally, no specific clothing requirements were asked to the users and no pre-selection of the users was performed.

Table 1 shows the comparison of the mean BR obtained through the proposed algorithm within the whole acquisition timestamp and the original forced rate. Almost all of the user were measured twice for each of the proposed rates and three of them were asked to repeat the measurements at a different frequency value.

As could be seen in the results, this first experiment proved the ability of the proposed algorithm to successfully retrieve the respiratory function of the users. To quantify the performance of the algorithm, the Root Mean Squared Error (RMSE) has been computed as

(6) $$RMSE=\frac{1}{N}\sqrt{\sum_{n=1}^{N}(BR_{n}-\hat{BR_{n}{)}^{2}}}$$

where $\hat{BR_{n}}$ accounts for the estimated BR obtained from the Video Signal (VS) and $BR_{n}$ accounts for the one obtained from the Ground Truth (GT). In this first case, the Ground Truth corresponds to the fixed frequency of the metronome. The acquisitions of this first experiment gave an overall RMSE of 0.18 breath/min, which is a sufficiently small error—according to the state-of-the-art algorithms—to first validate the algorithm and move forward to a more accurate analysis of the acquired signal during a second experiment.

**Figure 4:**
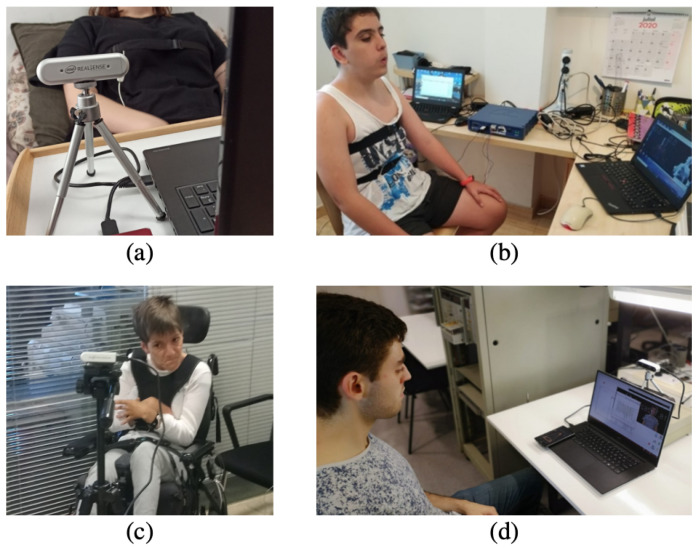
(**a**) Acquisition in a home environment with the user lying in bed. (**b**) Acquisition in a home environment with the user siting in a chair. (**c**) Acquisition in a healthcare environment with a disabled user. (**d**) Acquisition in the laboratory.

Despite the fact that all the acquisitions were carried out with static users, different poses and acquisition environments were also considered for validation purposes. Figure 4 shows pictures of the different poses and environments tested. Finally, it is interesting to note that different participant’s physical condition have been also considered.

**Figure 5:**
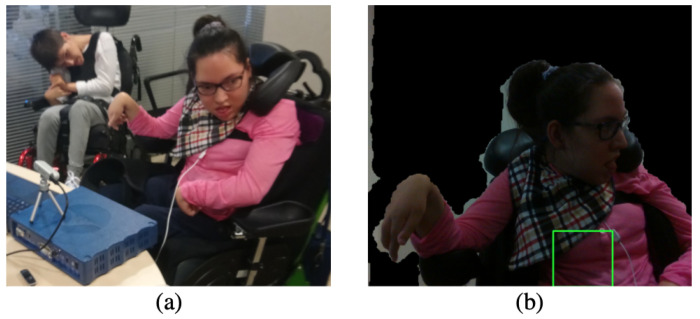
(**a**) Acquisition in a healthcare environment with a disabled user showing a non-standard pose. (**b**) Computer view during the execution of the proposed algorithm. The masked background could be observed as well as the ROI, which is marked in green.

Figure 5a shows another example of acquisition in a non-ambulatory environment and Figure 5b shows the corresponding computer view. As could be observed, there is no strict necessity to look directly towards the camera. The only condition is a visible face—or even half of it—and a (quasi)-static user. Involuntary movements due to users’ physical conditions, for example, in cerebral palsy users, have not shown a direct impact on the acquired signal.

### 3.2. Second Experiment: Multi ROI

**Figure 6:**
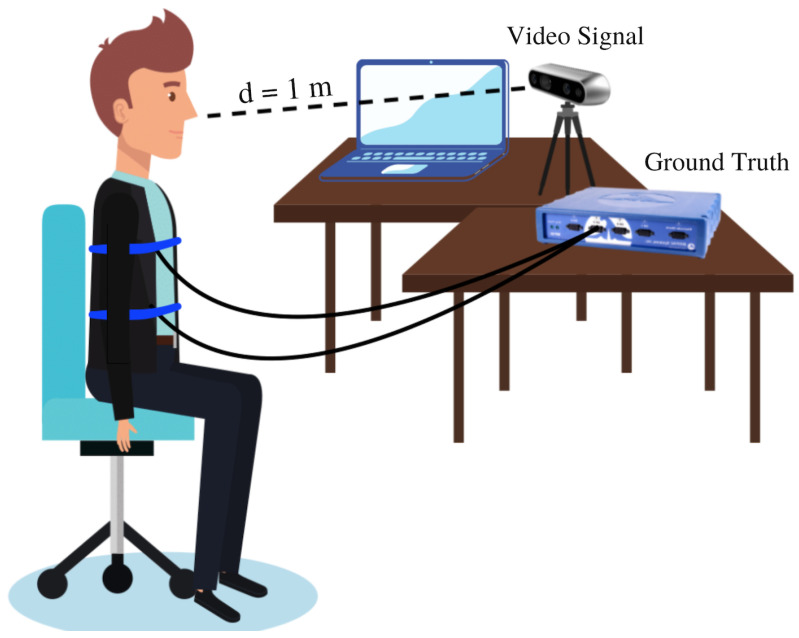
The user is at a distance of about 1 m to the camera and facing it. Meanwhile, the Ground Truth signal is acquired by direct contact with a medical device.

The second experiment was meant to carefully examine the acquired signal in individual inhalation–exhalation cycles, apart from the mean BR computation from the whole acquisition period, and to prove that multiple body regions can be simultaneously acquired. To do so, the respiratory signal of another set of 20 voluntary users, namely, U11-U30, was acquired by both the developed system for the Intel RealSense Depth Camera D435 (Video Signal) and the system *Biopac MP36* (Ground Truth). Afterwards, both signals were compared in order to validate and quantify the performance of the new proposed non-contact system. In this case, two ROIs were selected—thorax and abdomen—to be measured simultaneously proving the multi-target ability of the proposed algorithm. The ages of the participants ranged from 13 to 63 years old with an average age of 38.6 years. Again, all the participants provided their explicit consent. Each user was invited to sit on a chair at a mean distance of 1 m to the acquisition device. They were asked to face the camera, remain still and breathe naturally during the measurements. All the measurements were carried out indoors, but again in different scenarios, with a stable amount of light and during 60 s each. Figure 6 shows the described setup used in each of the acquisitions.

**Figure 7:**
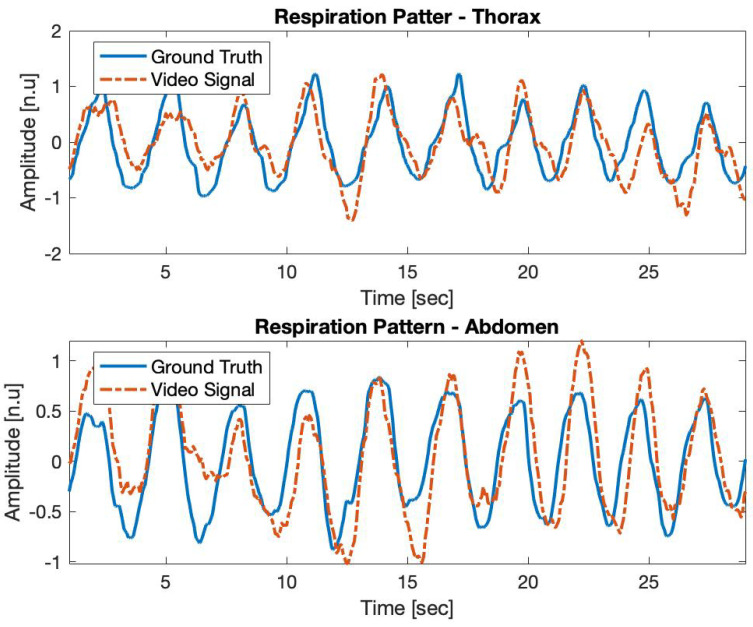
Comparison of the respiratory pattern obtained through the proposed algorithm (Video Signal) and the reference value (Ground Truth) during 30 s of continuous acquisition. The signals have been acquired at the thoracic (**top** figure) and abdominal (**bottom** figure) regions.

Once the measurement starts, the algorithm computes the thoracic and abdominal regions and starts a simultaneous acquisition. As mentioned, the developed system measures the position of the ROIs with respect to the camera via the depth channel. The Ground Truth signal is obtained by the *Biopac MP* system and it is acquired by using a piezoelectric sensor attached to a band that is placed at the ROIs computed by the algorithm. The principle of acquisition is mostly the same as with the camera since the piezoelectric sensor also measures the movements of those regions, but with respect to the band. Nevertheless, although the principle is the same, we cannot assure the same waveform in both cases. Figure 7 shows a comparison between the waveform of the Ground Truth and the one obtained through the Video Signal in two different body regions. It shows that, although the waveform presents deviations in terms of amplitude, the temporal relation regarding the peaks of the signal is maintained. Therefore, the acquired signal could be compared to the Ground Truth via the BR that is obtained by computing the time between the peaks of the signal. Any other parameter relying on the time-domain information could be also computed.

**Figure 8:**
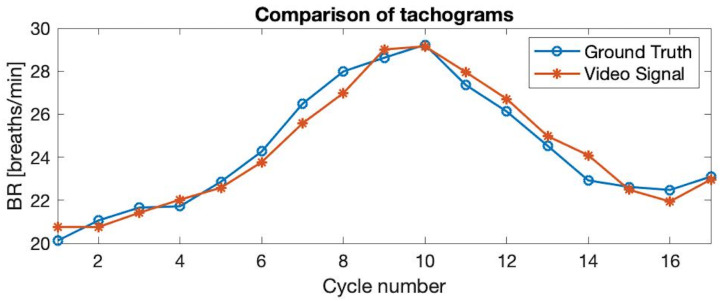
Comparison of the tachogram of the breath rate obtained through the Video Signal (blue line) with respect to the Ground Truth signal (orange line). It shows the ability of the proposed algorithm to follow sharp changes in the breath rate. In this case, from 20 breaths/min to 29 breaths/min, approximately.

Figure 8 shows a comparison of the Video Signal and the Ground Truth through the tachogram of the BR in both cases, showing that the temporal relation between the two signals can be compared.

In this case, the BR is computed relying on the breath-by-breath information, but also by averaging the values within windows of 5 inhalation–exhalation cycles.

**Figure 9:**
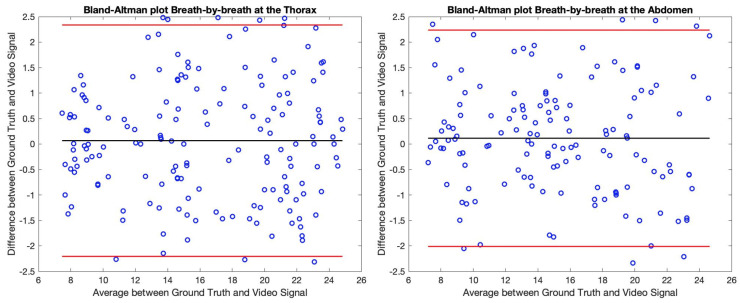
Bland–Altman representation of the thoracic (**left** image) and abdominal (**right** image) regions regarding the real-time assessment of the respiratory function. The black line shows the bias of the distribution while the red lines show the ±96% limits of agreement. Each individual pair of samples has been represented in blue.

**Figure 10:**
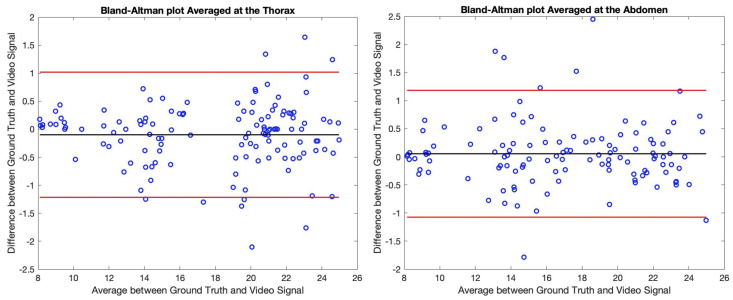
Bland–Altman representation of the thoracic (**left** image) and abdominal (**right** image) regions regarding the averaged values in windows of 5 cycles. For the averaged values, a first window of 5 respiratory cycles has been selected, then this window has been displaced one position with each new cycle. The black line shows the bias of the distribution while the red lines show the ±96% limits of agreement. Each individual pair of samples has been represented in blue.

The tachograms of all the participants have been analysed and the results have been collected into the Bland–Altman plots of Figure 9 and Figure 10. Concretely, these representations show the difference between the BR obtained trough the Video Signal and the Ground Truth with respect to their mean values regarding the breath-by-breath information (Figure 9), and also the averaged values (Figure 10).

By observing the Bland–Altman plots, one can easily notice that the BR estimation of the proposed algorithm closely coincides with the reference values. The real-time values have similar biases regarding the thoracic and abdominal measures while the ±96% limits of agreement are roughly between ±2 breaths/min. By averaging the obtained values in 5 cycles, the limits of agreement get reduced to almost ±1 breath/min. Concretely, regarding the measures at the thorax, the ±96% limits of agreement decrease from −2.21/2.34 breaths/min with a bias of 0.07 breaths/min to −1.22/1.02 breaths/min with a bias of −0.10 breaths/min comparing breath-by-breath information to the averaged values. Regarding the measures at the abdomen, the limits of agreement decrease from −2.01/2.24 breaths/min with a bias of 0.11 breaths/min to −1.07/1.18 breaths/min with a bias of 0.06 breaths/min.

Finally, the same BR information has been compared in terms of the mean value of each participant in the recorded periods of 60 s. Table 2 shows a comparison of the mean BR obtained through the proposed algorithm and the one computed considering the signal acquired by the system *Biopac MP36* regarding the breath-by-breath information. Table 3 shows the same information, but for the averaged values in windows of 5 inhalation–exhalation cycles. As could be observed in the results, two thoracic acquisitions were discarded due to periods of no acquisition within the considered timestamp, and three abdominal acquisitions were discarded because of a poorly acquired reference signal. We hypothesize that the periods of no acquisition occurred because of the saturation of the acquisition device, an unstable frame rate or even because of movement artifacts occluding the ROIs, while the problems with the reference signal are due to the sensor’s position at the abdomen.

Finally, to analyze the individual mean BR values obtained, the RMSE has been computed as in the first experiment. The acquisitions of this second experiment gave an overall RMSE of 0.21 breath/min in the breath-by-breath assessment and an overall RMSE of 0.13 breath/min in windows of 5 inhalation–exhalation cycles. These results show the feasibility of applying the proposed algorithm for a reliable assessment of the respiratory information.

## 4. Discussion

The experimental results obtained by the proposed non-contact assessment of the respiratory function in different parts of the body with an algorithm achieving fixed-time complexity O(1) are comparable to those reported in the literature. In particular, we have found a non-statistically significant bias of 0.07 breaths/min and the 96% limits of agreement of −2.21 to 2.34 breaths/min in the breath-by-breath assessment of the thoracic region. Similarly, in the abdominal region, we have found a bias of 0.11 breaths/min and the 96% limits of agreement of −2.01 to 2.24 breaths/min. Regardless of the region, the breath-by-breath assessment shows a mean RMSE of 0.21 breaths/min. Both the presented 96% limits of agreement and the RMSE can be reduced by averaging the obtained values in consecutive inhalation–exhalation cycles. This could be also a valid approach to obtain smaller errors despite of losing real-time information. For example, if one considers a normal and stable BR of 16 breaths/min, the proposed algorithm could display the breath-by-breath information every 3.75 s approximately in real-time as it should be the time between the last inhalation peak and its consecutive. In comparison, if one accesses the averaged information in two inhalation–exhalation cycles, a new value update will be resolved every 7.50 s with a smaller error. We have proved that the RMSE error could be reduced up to 0.13 breaths/min by considering five of those cycles. The presented results of the second experiment have been computed by comparing the acquired signal of the proposed system to the reference values obtained through the estimation performance of a medical device. Therefore, the accuracy has been experimentally quantified as the difference between the signal obtained by the proposed method and the reference one. We have found a maximum uncertainty of 2.34 breath/min in the real-time assessment, and that it can be reduced up to 1.22 breath/min if instead of estimating it for each respiratory cycle, an average of five cycles is considered. Finally, although the obtained error values in the two considered regions are similar, one should consider that some body regions present more mechanic movement than others as a result of the respiration when selecting the region under study, since the lack of movement will directly impact on the quality of the obtained results.

The novelty of the proposed method is to reduce the acquisition algorithm to a simple mean depth value computation of the target regions, getting rid of the processing of the traditional RGB channels that are prone to be more time-consuming. Instead, the Viola–Jones algorithm is applied to the RBG stream to locate the face of the user in the scene and automatically compute the regions in which the measurement is going to be performed according to the physiology of the user. Note that the main acquisition is performed just with the information of the depth channel, aside from the detection and location of the ROIs. Therefore, multiple ROIs can be added to the simultaneous measurement without altering the computational flow, and real-time values can be also registered. In this article, we have presented the results of the synchronous assessment of both the thoracic and abdominal regions. Nevertheless, these regions could be changed by direct modification of Equations (1)–(4) to assess the respiratory signal in any other part of the body. For example, the comparison of the left and right movements of the chest in order to see if there is an homogeneous expansion of the thorax could be also of medical interest. Experimental tests have validated that the developed system works correctly if the distance from the user to the camera is up to 1.5 m, where the background mask is applied. In fact, regardless of the background mask, the proposed method has shown similar accuracy values up to 2 m of distance between the user and the acquisition device. The measurements, by using the depth sensor, are less affected by changes in light intensity, so its use is possible in non-hospital environments. The most important factor for its operation is a correct recognition of the user’s face, as the rest of the configuration parameters are automatically adjusted based on this first detection.

Regarding the state-of-the-art, several non-contact approaches have been proposed in the literature for the estimation of the respiratory function based on the movement of the body regions when breathing. Among others, Siam et al. extracted the respiratory function from the automatically detected chest region by using an IR camera with an averaged RMSE of 0.464 breaths/min [30]. However, no breath-by-breath information could be retrieved as the BR estimation was done by averaging the inhalation–exhalation cycles of the entire recordings. In addition, one single and static ROI was considered and the algorithm relied on the integral of the ROI instead of in the mean depth value. Massaroni et al. used a single RGB camera to extract the respiratory pattern from intensity variations of reflected light at the level of the collar bones and above the sternum [3]. In this case, they extracted breath-by-breath information with an error of 0.55 breaths/min as its best performance and 1.53 breaths/min in lower resolution, considering again one single and static region. In addition, they processed the three RGB channels individually, not being able to provide the assessment in real-time. Finally, Benetazzo et al. also proposed a method for extracting the respiratory pattern by means of a depth sensor in an automatically located chest region [29]. Nevertheless, a complex postprocessing of the signal was proposed for the extraction of the valuable information making impossible the real-time assessment. In addition, in this case, only one single and static ROI was considered at a time and the algorithm could not be externally modified to select the desired region, i.e., the monitoring could be only performed at the chest. Finally, our study overcomes by far the variety and number of users considered. In comparison, our proposed method achieves smaller errors and multi-point assessment due to the reduced complexity of processing one single depth channel. To our best knowledge, the non-contact and simultaneous multi-region estimation of the respiratory function directly implemented on the depth channel and using the RGB channels aside to adapt the algorithm to the user has not been proposed before. In addition, the errors obtained in both the breath-by-breath assessment and the averaged estimations make the proposed method suitable for remote health tracking as it overcomes the performance of the state-of-the-art algorithms.

## 5. Conclusions

In this paper, we present a non-contact system to simultaneously estimate the respiratory function in multiple parts of the body. The respiratory function is estimated based on the breathing movement of those target regions with respect to the camera. Concretely, these movements are captured by the depth channel of an Intel Real Sense D435 camera. However, any other RGB-D camera-based technology with a depth module of the same characteristics and separate RGB channels could also be used. The non-contact and simultaneous multi-region estimation, up to 10 ROIs, of the respiratory function directly implemented on the depth channel, using the RGB channels aside to adapt the algorithm to the user is newly proposed in this work. A first experiment has been conducted for validation purposes considering different scenarios, different body poses and body types (body sizes and skin tones), physical conditions and clothing, already giving competent results regarding the state-of-the-art. In addition, a more accurate second experiment has been performed to closely compare the acquired signal to the reference one regarding both breath-by-breath and averaged assessments. The errors obtained in both the breath-by-breath assessment, from −2.21 to 2.34 breaths/min in the worst case, and the averaged estimations, from −1.22 to 1.18 breaths/min in the worst case, make the proposed method suitable for remote health tracking. The overall RMSE errors of 0.21 breaths/min in the breath-by-breath assessment and 0.13 breaths/min in the averaged estimation also overcome the state-of-the-art performances. The proposed system enables breath monitoring in static situations using a highly accurate and low-cost system, which makes it suitable for non-hospital applications and periodic monitoring of respiratory function.

## Figures and Tables

**Table 1:** Table of the mean breath rate of each user obtained through the proposed algorithm and the original forced rate.

User	Age	Gender	Forced Rate	Estimation
			[breaths/min]	[breaths/min]
U1	66	Male	12	12.06
				11.97
U2	60	Female	14	13.98
				14.06
U3	21	Female	15	15.05
				14.96
U4	54	Male	15	15.13
				-
U5	27	Male	16	16.09
				16.05
U6	24	Female	18	18.00
				18.13
U7	22	Male	18	18.19
				-
			12	11.42
				12.03
U8	23	Female	20	20.43
				-
U9	22	Female	22	22.21
				22.01
			18	18.09
				18.24
U10	21	Male	25	25.00
				24.97
			15	15.11
				15.00

**Table 2:** Table of the mean breath rate of each user obtained by the breath-by-breath information (in breaths/min) of the Video Signal (VS) in comparison to the Ground Truth (GT).

User	Age	Gender	VS	GT	VS	GT
			Thorax	Thorax	Abdomen	Abdomen
U11	50	Male	24.49	24.44	24.18	23.99
U12	18	Male	28.44	28.19	29.00	28.46
U13	13	Male	21.88	21.34	19.70	19.81
U14	50	Female	23.22	23.32	23.51	23.44
U15	48	Female	21.84	21.72	21.54	22.32
U16	36	Male	13.98	13.80	14.70	14.83
U17	54	Male	10.91	10.68	9.03	9.13
U18	23	Female	14.37	14.37	14.38	14.45
U19	50	Male	9.66	9.51	9.80	9.72
U20	56	Male	8.27	8.27	8.21	8.17
U21	21	Male	9.48	9.53	9.54	9.60
U22	14	Male	12.71	12.68	11.24	11.26
U23	49	Male	8.35	8.18	8.41	8.42
U24	61	Male	21.66	21.58	21.88	21.74
U25	22	Female	-	-	17.42	17.54
U26	48	Male	20.58	20.64	-	-
U27	63	Female	-	-	13.85	13.81
U28	23	Male	20.84	20.99	-	-
U29	50	Male	14.48	14.54	-	-
U30	22	Female	15.79	15.70	15.86	15.93

**Table 3:** Table of the mean breath rate of each user in (breaths/min) obtained by averaging the Video Signal (VS) in 5 inhalation-exhalation cycles in comparison to the Ground Truth (GT) also averaged.

User	Age	Gender	VS	GT	VS	GT
			Thorax	Thorax	Abdomen	Abdomen
U11	50	Male	24.30	24.34	24.30	24.30
U12	18	Male	28.15	28.31	28.02	28.17
U13	13	Male	20.15	20.38	19.40	19.32
U14	50	Female	23.14	23.18	23.24	23.22
U15	48	Female	21.52	21.42	21.13	21.57
U16	36	Male	12.35	12.48	14.02	14.07
U17	54	Male	9.72	9.67	9.17	8.88
U18	23	Female	13.95	14.19	14.38	14.45
U19	50	Male	9.10	9.04	9.22	9.23
U20	56	Male	8.24	8.19	8.25	8.23
U21	21	Male	9.51	9.39	9.32	9.38
U22	14	Male	11.78	11.91	10.95	10.88
U23	49	Male	8.25	8.12	8.40	8.39
U24	61	Male	22.08	21.97	21.90	21.93
U25	22	Female	-	-	17.01	17.01
U26	48	Male	20.59	20.65	-	-
U27	63	Female	-	-	13.85	13.81
U28	23	Male	20.77	20.79	-	-
U29	50	Male	14.41	14.55	-	-
U30	22	Female	15.93	15.72	15.86	15.93

## Data Availability

The data presented in this study are available on request from the corresponding author.
